# The impacts of COVID-19, meteorology, and emission control policies on PM_2.5_ drops in Northeast Asia

**DOI:** 10.1038/s41598-020-79088-2

**Published:** 2020-12-17

**Authors:** Yoon-Hee Kang, Seunghee You, Minah Bae, Eunhye Kim, Kyuwon Son, Changhan Bae, Yoonha Kim, Byeong-Uk Kim, Hyun Cheol Kim, Soontae Kim

**Affiliations:** 1 Environmental Research Institute, Ajou University, Suwon, Republic of Korea; 2 Department of Environmental and Safety Engineering, Ajou University, Suwon, Republic of Korea; 3 Emission Inventory Management Team, National Air Emission Inventory and Research Center, Ministry of Environment, Cheongju, Republic of Korea; 4 Georgia Environmental Protection Division, Atlanta, GA 30354 USA; 5 Air Resources Laboratory, National Oceanic and Atmospheric Administration, College Park, MD 20740 USA; 6 Cooperative Institute for Satellite Earth System Studies, University of Maryland, College Park, MD 20740 USA

**Keywords:** Climate sciences, Environmental sciences, Environmental social sciences

## Abstract

In January 2020, anthropogenic emissions in Northeast Asia reduced due to the COVID-19 outbreak. When outdoor activities of the public were limited, PM_2.5_ concentrations in China and South Korea between February and March 2020 reduced by − 16.8 μg/m^3^ and − 9.9 μg/m^3^ respectively, compared with the average over the previous three years. This study uses air quality modeling and observations over the past four years to separate the influence of reductions in anthropogenic emissions from meteorological changes and emission control policies on this PM_2.5_ concentration change. Here, we show that the impacts of anthropogenic pollution reduction on PM_2.5_ were found to be approximately − 16% in China and − 21% in South Korea, while those of meteorology and emission policies were − 7% and − 8% in China, and − 5% and − 4% in South Korea, respectively. These results show that the influence on PM_2.5_ concentration differs across time and region and according to meteorological conditions and emission control policies. Finally, the influence of reductions in anthropogenic emissions was greater than that of meteorological conditions and emission policies during COVID-19 period.

Since the first reported case of the novel coronavirus disease (COVID-19, a respiratory syndrome caused by SARS-CoV-2 infection) in Wuhan, China in December 2019^1,2^, COVID-19 has spread worldwide and as of June 21, 2020 the World Health Organization (WHO) reported that 8,708,008 people have been infected^3^. China, where the outbreak of COVID-19 was first reported, and South Korea, a neighboring country, declared national emergencies concerning the rapid spread of COVID-19 infections in mid-January 2020. Most economic activities were limited until restrictions were eased in China and South Korea in April and May 2020, respectively. The decrease in economic activities, including reduced traffic since January 2020, has led to the reduction of anthropogenic emissions caused by outdoor activities of the general public^4,5^. Over the course of the COVID-19 outbreak, reductions in air pollutant concentrations such as PM_2.5_ (particles with a diameter of 2.5 μm or less), which posed serious threats with high environmental risks in Northeast Asia, have also been reported^6,7^.

In Northeast Asia, high concentration of PM_2.5_ and related haze events frequently occurred in winter and early spring, mainly in China and South Korea where large amounts of air pollutant emissions are concentrated^8–11^. From December to March for the three years prior to the outbreak of COVID-19 (December 2016 to March 2017, December 2017 to March 2018, and December 2018 to March 2019, defined as the non-COVID-19 period), the PM_2.5_ concentrations observed on the surface in China and South Korea were 62.2 μg/m^3^ and 31.6 μg/m^3^, respectively. In contrast, the PM_2.5_ concentrations observed from December 2019 to March 2020 (hereinafter defined as the COVID-19 period) were 50.0 μg/m^3^ and 24.5 μg/m^3^ respectively. They were reduced by 19.6% and 22.6% compared with the concentrations observed during the non-COVID-19 period.

When comparing the trend of PM_2.5_ concentrations observed from December to March from 2016 to 2020 in China and South Korea (Fig. 1), China showed around a − 2.35 μg/m^3^/month reduction in the PM_2.5_ concentration in the non-COVID-19 period prior to the COVID-19 outbreak. In contrast, South Korea showed a + 0.39 μg/m^3^/month increase in PM_2.5_ concentration. However, the PM_2.5_ concentration showed a drastic decrease after the COVID-19 outbreak. During the period of COVID-19, the concentration in China showed a sharp decrease of − 9.82 μg/m^3^/month. While the concentration trend of South Korea showed a slight increase prior to the COVID-19 period, it decreased by − 1.57 μg/m^3^/month after the outbreak.

**Figure 1:**
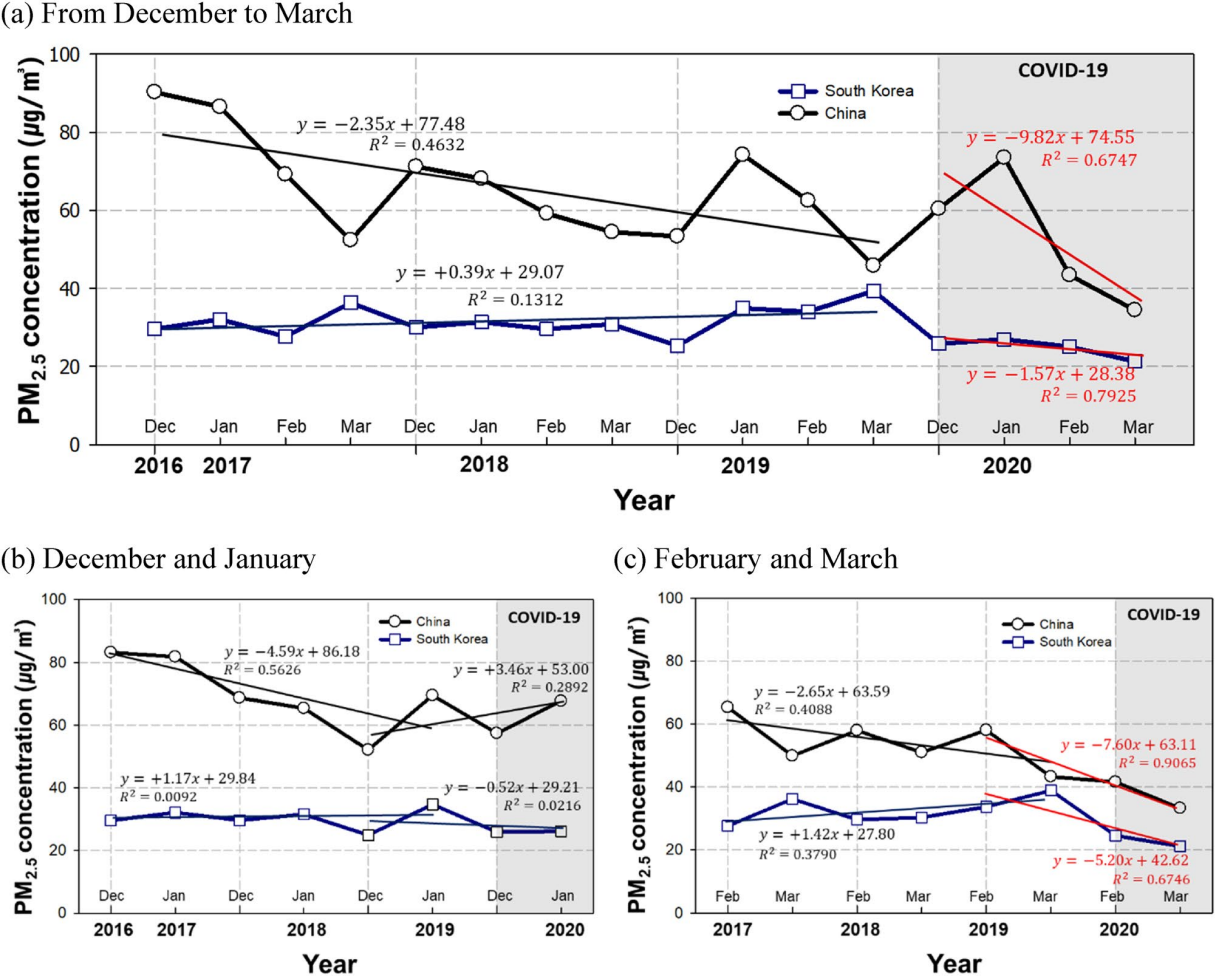
Monthly average change trends of PM_2.5_ concentration observed in China and South Korea in 2016 to 2020. (**a**) From December to March (**b**) December and January, and (**c**) February and March. The blue and black lines represent the observed PM_2.5_ concentrations in South Korea and China, respectively.

We examined the changes in PM_2.5_ concentration by into a first half (December and January) and second half (February and March), focusing on the period when the COVID-19 confirmed patients surged in China and South Korea (Fig. 1b,c). In the first half, prior to the COVID-19 outbreak, the concentration of PM_2.5_ decreased by − 4.59 μg/m^3^/month in China and increased by + 1.17 μg/m^3^/month in South Korea. In the early stage of COVID-19 outbreak, the concentration even increased (+ 3.46 μg/m^3^/month) in China or showed a slight decrease (− 0.52 μg/m^3^/month) in South Korea. In the second half, when outdoor activities were limited, both China and South Korea showed a clearly declining trend of PM_2.5_ concentration (China: − 7.60 μg/m^3^/month, South Korea: − 5.20 μg/m^3^/month). In the second half of the COVID-19 period, the regression slope for PM_2.5_ concentration decreased by 5.0 µg/m^3^/month in China and by 6.6 µg/m^3^/month in South Korea compared with the slope for PM_2.5_ concentration observed during the non-COVID-19 period.

The generation and dissipation of air pollutants undergo highly complex chemical/physical processes according to various atmospheric conditions such as meteorological conditions and emissions, and the final concentration of pollutants in the atmosphere is determined from these processes^12^. Therefore, it is difficult to identify that the reduction in the observed PM_2.5_ concentrations during the COVID-19 period was directly influenced by the restrictions on anthropogenic activity. In response to the serious air pollution problems observed in the Northeast Asian region, governments in the region have been implementing policies to reduce emissions since early 2010, and as a result steady reductions in the concentrations of air pollutants, with priority given to primary pollutants, have been observed^13–17^. Moreover, owing to the recent climate change in Northeast Asia and changes in synoptic weather conditions, fluctuations in air pollutant concentrations have increased^18,19^. Because of this, it is difficult to determine the direct influence of the anthropogenic effects of COVID-19 on PM_2.5_ concentrations observed in February and March 2020, and air pollutant concentration reduction trends during the COVID-19 period have only been confirmed by data such as surface observations and satellite images in most studies^7,20,21^. Alternatively, air quality modeling has been performed after assuming reduced emissions and the influences on the air pollutant concentration estimated^5,22,23^. Until now, there has been no study to quantitatively present the reduction of observed air pollutant concentrations influenced by COVID-19, separated from the influences of meteorological conditions and emission reduction policies. This study first aimed to distinguish the influences of meteorological conditions and emissions expected to be related to the PM_2.5_ concentration reduction observed in the Northeast Asian region during the COVID-19 period. Secondly, the influences of emission reductions following emission control policies were separated and the results were presented quantitatively. To this end, meteorology-air quality modeling was performed for December to March for the past 4 years (2016–2020), including the COVID-19 period, and compared with the observed PM_2.5_ concentrations.

Figure 2 shows the normalized anomaly from PM_2.5_ concentrations observed and simulated during the non-COVID-19 and COVID-19 periods in China and South Korea. Air quality modeling was performed by inputting fixed anthropogenic emissions that do not account for annual changes in Northeast Asia. We used average PM_2.5_ concentration during the non-COVID-19 period for each observation and simulation for the required average value for anomaly calculation. The observed PM_2.5_ concentrations and the simulated PM_2.5_ concentrations showed differences because of the uncertainty of the input data and the parametrization process in the model^24^, but the temporal change trends of the simulated and observed PM_2.5_ concentration were similar, and the average prediction accuracy of the meteorology and air quality simulation results were reliable (R > 0.90 of air temperature at an altitude of 2 m and wind speed at an altitude of 10 m, R > 0.89 of PM_2.5_ concentration simulated during the non-COVID-19 period, see Table S1 and Figs. S3 to 5 in Supplement for meteorology and air quality simulation results). For the non-COVID-19 period, the observed and simulated PM_2.5_ showed a similar level of anomaly, and the trend of concentration change over time was also similar. However, for the COVID-19 period, the difference between the observed and the simulated anomaly increased (the average value of the difference between the non-COVID-19 period observation and the simulated anomaly: China 0.0% and South Korea 3.4%, the average value of the difference between the COVID-19 period observation and the simulated anomaly: China − 17.2%, and South Korea − 32.3%). In particular, the difference between observation-simulation anomaly for both China and South Korea were large, − 31.8% and − 57.1%, respectively in February. In other words, the observed concentration of PM_2.5_ in the COVID-19 period decreased compared with that in the non-COVID-19 period in the Northeast Asia region, but the model predicted the PM_2.5_ concentration to slightly decrease (China) or even increase (South Korea). The main reason for the large differences in observation-simulation anomaly during the COVID-19 period is the failure of the model to reflect reductions in anthropogenic emissions during the COVID-19 period.

**Figure 2:**
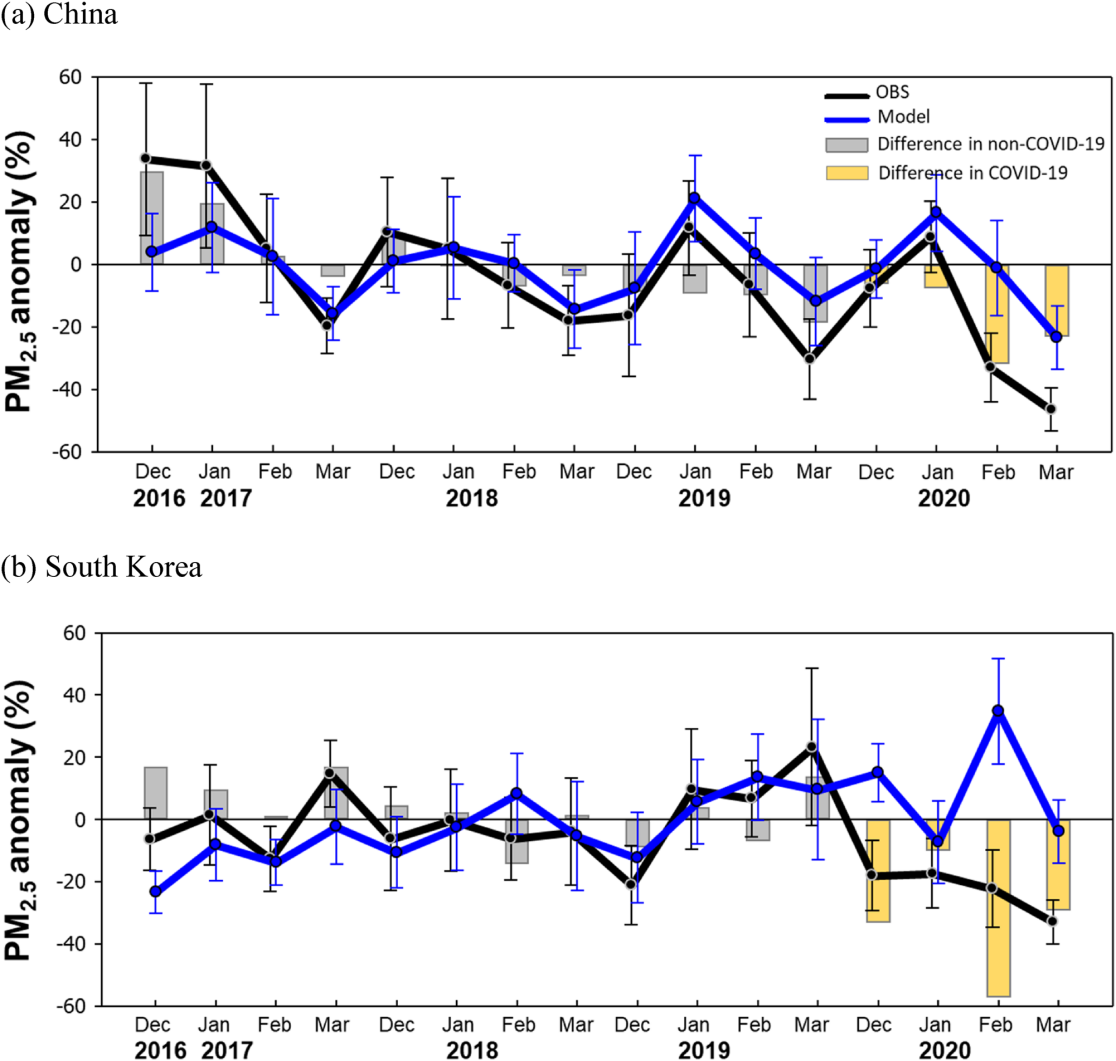
Difference between the observation-simulation anomalies of PM_2.5_ in China and South Korea from December to March in 2016 to 2020. Average concentrations of simulated PM_2.5_ were 58.9 μg/m^3^ for China and 26.2 μg/m^3^ for South Korea. Average concentrations of observed PM_2.5_ were 62.2 μg/m^3^ for China and 31.6 μg/m^3^ for South Korea. The blue line represents the anomaly for the simulated concentration and the black line represents the anomaly for the observed concentration. The gray and yellow bars represent the non-COVID-19 period and COVID-19 period, respectively.

Figure 3 shows the trends in PM_2.5_ concentration for the first half (December to January) and the second half (February to March) of the COVID-19 period for China and South Korea, and the quantitative influences of meteorological conditions, emission control policies, and COVID-19 on the PM_2.5_ concentration changes (Refer to methods section). Both China and South Korea show a steeper decline in the concentration of PM_2.5_ in the second half, when the COVID-19 infected patients surged and outdoor activities were more limited, compared with the earlier two months right after the outbreak of COVID-19. In China, compared with the PM_2.5_ concentration of the non-COVID-19 period, the average concentration of PM_2.5_ decreased by − 7.6 µg/m^3^ in the first half and − 16.8 μg/m^3^ in the second half. In South Korea, the PM_2.5_ concentration decreased by − 4.4 μg/m^3^ for the first half and − 9.9 μg/m^3^ for the second half of COVID-19 period.

**Figure 3:**
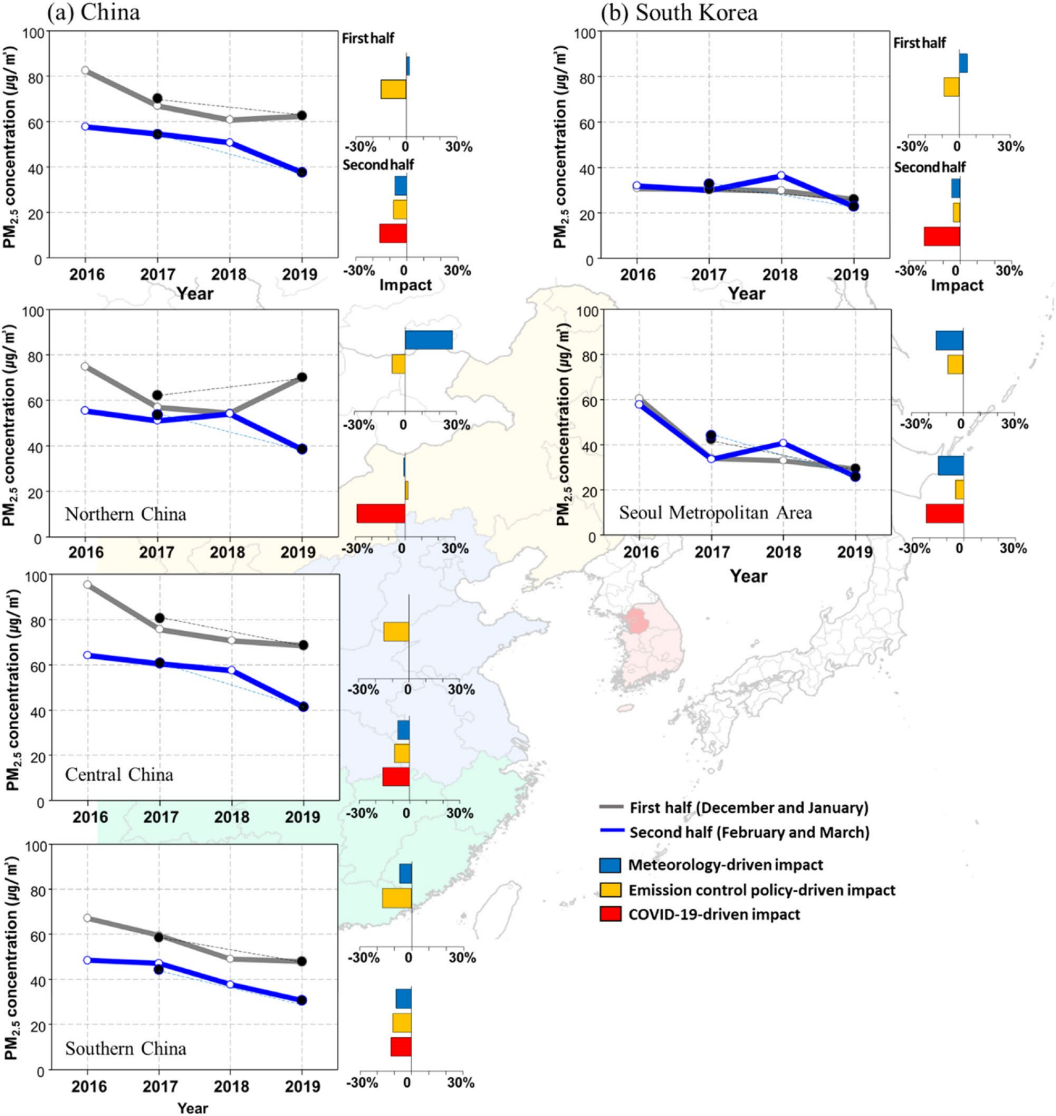
Trends of period-mean PM_2.5_ concentrations and meteorology, and emission control policy and COVID-19-driven impacts on the change of PM_2.5_ concentrations in the first half (December and January) and the second half (February and March) during 2016–2019 over China (including North, Central and South China) and South Korea (including SMA). The gray and blue lines indicate the PM_2.5_ trends in the first and second halves, respectively. The black dots are the averaged PM_2.5_ concentrations in the non-COVID-19 period (2016–2018) and the COVID-19 period (2019). The blue, yellow, and red bars indicate the meteorology, emission control policy and COVID-19-driven impacts on PM_2.5_ changes. The yellow, blue, and green regions are Northern, Central, and Southern China. The red region is SMA in South Korea (pink). The map was generated using Interactive Data Language version 8.7.0 (Harris Geospatial Solutions, http://harrisgeospatial.com) with Global Administrative Areas (http://gadm.org) map data.

Meteorological conditions in China and South Korea contributed to the increase in PM_2.5_ concentration in the first half of the COVID-19 period (2% in China and 5% in South Korea), but a decrease in PM_2.5_ concentration in the second half (− 7% in China and − 5% in South Korea). In particular, in northern China, the concentration of PM_2.5_ in the first half of the COVID-19 period increased by approximately 8.0 µg/m^3^ compared with that during the non-COVID-19 period, where the meteorological conditions played a significant role of + 28% (17.1 µg/m^3^). The average wind speed and relative humidity in northern China in December 2019 and January 2020 were 2.3 ms^−1^ and 69.6%. When comparing these to the non-COVID-19 period, the wind speed was 10.6% lower, and relative humidity 6.7% higher (see Fig. S6 of Supplement). During the first half of the COVID-19 period, the meteorological conditions (i.e., decreased surface wind speed and increased relative humidity) in the Northeast Asia region limited the dispersion of air pollutants, including PM_2.5_, leading to an increase in the air pollutant concentrations^14,25^.

In contrast, the emission control policies in China and South Korea contributed to a reduction of PM_2.5_ concentration in both the first and the second half of the COVID-19 period. The influences of emission control policies on changes in PM_2.5_ concentrations were calculated to be − 15% (− 10.5 μg/m^3^) and − 8% (− 4.2 μg/m^3^) in China, and − 9% (− 2.9 μg/m^3^) and − 4% (− 1.3 μg/m^3^) in South Korea in the first and second half, respectively. Compared with the first half, the reduction of PM_2.5_ concentration due to policy in the second half fell to 40% in China and 44% in South Korea. Interestingly, in northern China, emission reduction policy contributed to a decrease of − 8% for PM_2.5_ concentration in the first half but an increase of 2% in the second half. This is due to the trend of change in the simulated PM_2.5_ concentration is larger than the observed PM_2.5_ concentration with a relatively large prediction error (RMSE 26.3 μg/m^3^, R 0.78, see Table S1 in Supplement) during the second half of northern China compared with other regions.

Lastly, the influence of anthropogenic emission reductions due to COVID-19 on PM_2.5_ concentrations calculated for the second half of COVID-19 were − 16% (− 9.0 μg/m^3^) for China (− 29% in the northern region, − 16% in the central region, and − 12% in the southern region) and − 21% (− 7.0 μg/m^3^) for South Korea (− 22% in Seoul Metropolitan Area (SMA)). In February to March 2020, when a distinct reduction was expected, these figures show that the influences of anthropogenic emissions reduction due to social distancing during the COVID-19 period in Northeast Asia were greater than that of meteorological conditions or emission control policies on the PM_2.5_ concentration reduction.

Our study used the observed and simulated PM_2.5_ concentrations from December to March for the past four years, including the COVID-19 period, to distinguish the influences of meteorological conditions and emissions on PM_2.5_ concentrations in the Northeast Asian region, and to separate the influences of anthropogenic emission reduction due to COVID-19 from national emission control policies. Note that it is assumed that influences of meteorology and emissions on the PM_2.5_ concentrations are independent to each other for the simple estimates in this study. Nevertheless, we believe that the method of separating the impacts of meteorology, emission control policy and COVID-19 presented in this study may be an effective way to evaluate the effectiveness of air quality regulations.

In Northeast Asia, especially China and South Korea, the severe PM_2.5_ pollution frequently occurred in winter and early spring, which draws a high public interest in air quality. In these countries, various emission reduction policies are continuously implemented to reduce the national PM_2.5_ levels. For example, the Chinese government actively implements the Air Pollution Prevention and Control Action Plan (2013–2017). In South Korea, the Seasonal Fine Dust Management System is implemented to mitigate the intensity and frequency of high PM_2.5_ concentrations from December to March. In this study, we have noticed to what extent the emission reductions are required to achieve a desired level of air quality over Northeast Asia. So far, policy-driven air quality managements have been effective to improve PM_2.5_ concentrations over Northeast Asia. However, significant emission reductions of PM_2.5_ and its precursors are inevitable to achieve an air quality level mutually agreeable by nations in the Northeast Asian region. More collaborative efforts of the community sharing airshed can accelerate our air quality improvement over the region.

## Methods

To clearly confirm the impact of COVID-19 on PM_2.5_ concentration, based on the period when the social distancing was actively implemented due to the spread of COVID-19 since the end of January in 2020, analysis was confirmed by dividing the period into a first half (December 2019 to January 2020) and second half (February to March 2020). For the non-COVID-19 period to be compared with the COVID-19 period, December to March for the previous 3 years (2016: December 2016 to March 2017, 2017: December 2017 to March 2018, 2018: December 2018 to March 2019) was chosen.

The PM_2.5_ concentration change for the COVID-19 period in Northeast Asia was calculated by comparing the average PM_2.5_ observed concentrations for the non-COVID-19 period and the COVID-19 period, respectively. The PM_2.5_ observed concentrations used are provided by the China National Environmental Monitoring Center (CNEMC) of China and Air Korea of South Korea. In addition, for the meteorological observation data for the analysis of the regional meteorological conditions, data from the Meteorological Assimilation Data Ingest System (MADIS) station, located in China and South Korea, were used. For quantitative analysis on the cause of PM_2.5_ concentration change over the COVID-19 period, the Community Multiscale Air Quality (CMAQ) model (ver.4.7.1) was used to predict air pollutant concentration in Northeast Asia including PM_2.5_ concentration. The modeling area consisting of Northeast Asia (174 × 128 grids with 27-km grid spacing as shown in Fig. S1) was classified into 6 regions (China, Northern China, Central China, Southern China, South Korea, and Seoul Metropolitan Area) to present the study results. The Weather Research Forecast (WRF) model (ver.3.9.1) developed by the National Center for Atmospheric Research (NCAR) was used to generate meteorological input data for the CMAQ model. The National Center for Environmental Prediction (NCEP) Global Forecast System (GFS) data on 1.0° × 1.0° grids gathered every 6 h was used for the initial condition and boundary condition of the meteorological simulation. For the anthropogenic emissions in Northeast Asia, CREATE 2015 Emission Inventory was input to the Sparse Matrix Operator Kernel Emissions (SMOKE) modeling system (ver.3.1) to convert to the CMAQ-ready emission input, and the land cover and vegetation data were input to the Model of Emissions of Gases and Aerosols from Nature (MEGAN) model (ver.2.1) for the estimation of biogenic emission.

**Figure 4:**
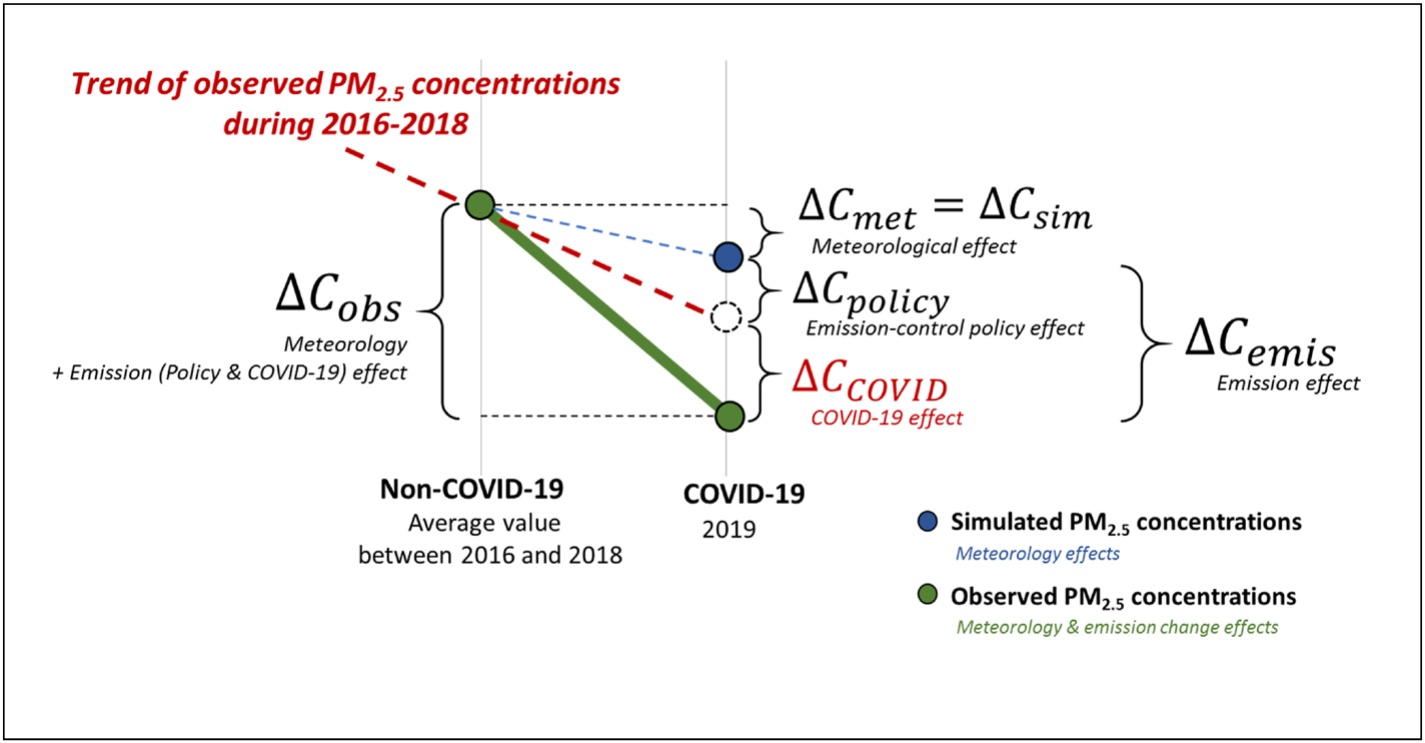
Schematic for the method of separating the impact of COVID-19 from meteorological and emissions control policies on the PM_2.5_ concentration change in Northeast Asia during the COVID-19 period.

CMAQ modeling for non-COVID-19 and COVID-19 periods was performed by entering fixed anthropogenic emissions with no annual change to determine the impact of meteorological conditions on PM_2.5_ concentration changes during the COVID-19 period in the Northeast Asia region (Fig. 4). The difference between the simulated PM_2.5_ concentrations in the non-COVID-19 period and the COVID-19 period ($\Delta {C}_{sim}$) did not take into account the emission changes from 2016 to 2020 (e.g., changes in emissions due to emission control policies for each country, or emission changes from issues such as COVID-19). Therefore, $\Delta {C}_{sim}$ can be seen as PM_2.5_ concentration change according to the meteorological changes ($\Delta {C}_{met}$).

In the next step, PM_2.5_ change due to the total change in emissions during the COVID-19 period ($$\Delta {C}_{emis}$$) was calculated by subtracting $$\Delta {C}_{met}$$ from the observed PM_2.5_ concentration change ($$\Delta {C}_{obs}$$). $$\Delta {C}_{emis}$$ includes not only the PM_2.5_ concentration change due to emission control policy ($$\Delta {C}_{policy}$$), but also anthropogenic emission reduction effects from social distancing during the COVID-19 period ($$\Delta {C}_{COVID}$$). To distinguish the two effects, $$\Delta {C}_{policy}$$ was calculated from the difference between the observed PM_2.5_ concentration change slope ($${Slope}_{O})$$ and the simulated PM_2.5_ concentration change slope ($${Slope}_{M})$$ during the non-COVID-19 period. $${Slope}_{M}$$ is the corrected slope calculated by multiplying the ratio of observed concentration and simulated concentration $$({\alpha }_{obs}= {C}_{obs}/{C}_{mod})$$ to the simulated PM_2.5_ concentration change slope to take into account the difference between observation and simulation. This was regarded as the PM_2.5_ concentration change slope by the meteorological changes.

Finally, for the second half of the COVID-19 period (February to March, 2020) when the impact of COVID-19 was clearly visible, the PM_2.5_ concentration change due to the COVID-19 outbreak ($\Delta {C}_{COVID}$) was obtained by subtracting $\Delta {C}_{policy}$ from $\Delta {C}_{emis}$.

## Supplementary information

Supplementary Information.
