# Supplementary material for: The impacts of COVID-19, meteorology, and emission control policies on PM2.5 drops in Northeast Asia
Source: Sci Rep. 2020 Dec 17;10:22112. doi: 10.1038/s41598-020-79088-2 (PMC7747715; doi:10.1038/s41598-020-79088-2)
Supplement: Supplementary file 1 — Supplementary Information. [file 41598_2020_79088_MOESM1_ESM.docx]

*Supplement of*

**The impacts of COVID-19, meteorology, and emission control policies on PM_2.5_ drops in Northeast Asia**

Yoon-Hee Kang^1)^, Seunghee You^2)^, Minah Bae^2)^, Eunhye Kim^2)^, Kyuwon Son^2)^, Changhan Bae^3)^, Yoonha Kim^1)^, Byeong-Uk Kim^4)^, Hyun Cheol Kim^5),6)^, Soontae Kim^2),^*

*^1)^ Environmental Research Institute, Ajou University, Suwon, Republic of Korea*

*^2)^ Department of Environmental and Safety Engineering, Ajou University, Suwon, Republic of Korea*

*^3)^ Emission Inventory Management Team, National Air Emission Inventory and Research Center, Ministry of Environment, Cheongju, Republic of Korea*

*^4)^ Georgia Environmental Protection Division, Atlanta, GA, 30354, USA*

*^5)^ Air Resources Laboratory, National Oceanic and Atmospheric Administration, College Park, MD, 20740, USA*

*^6)^ Cooperative Institute for Satellite Earth System Studies, University of Maryland, College Park, MD, 20740, USA*

^*^Corresponding author

E-mail: [soontaekim@ajou.ac.kr](mailto:soontaekim@ajou.ac.kr)

***Daily average change trends of PM_2.5_ concentrations during the non-COVID-19 and the COVID-19 periods***

Fig. S1 Daily average change trends of PM_2.5_ concentration observed in China and South Korea in December, 2016 to March, 2020


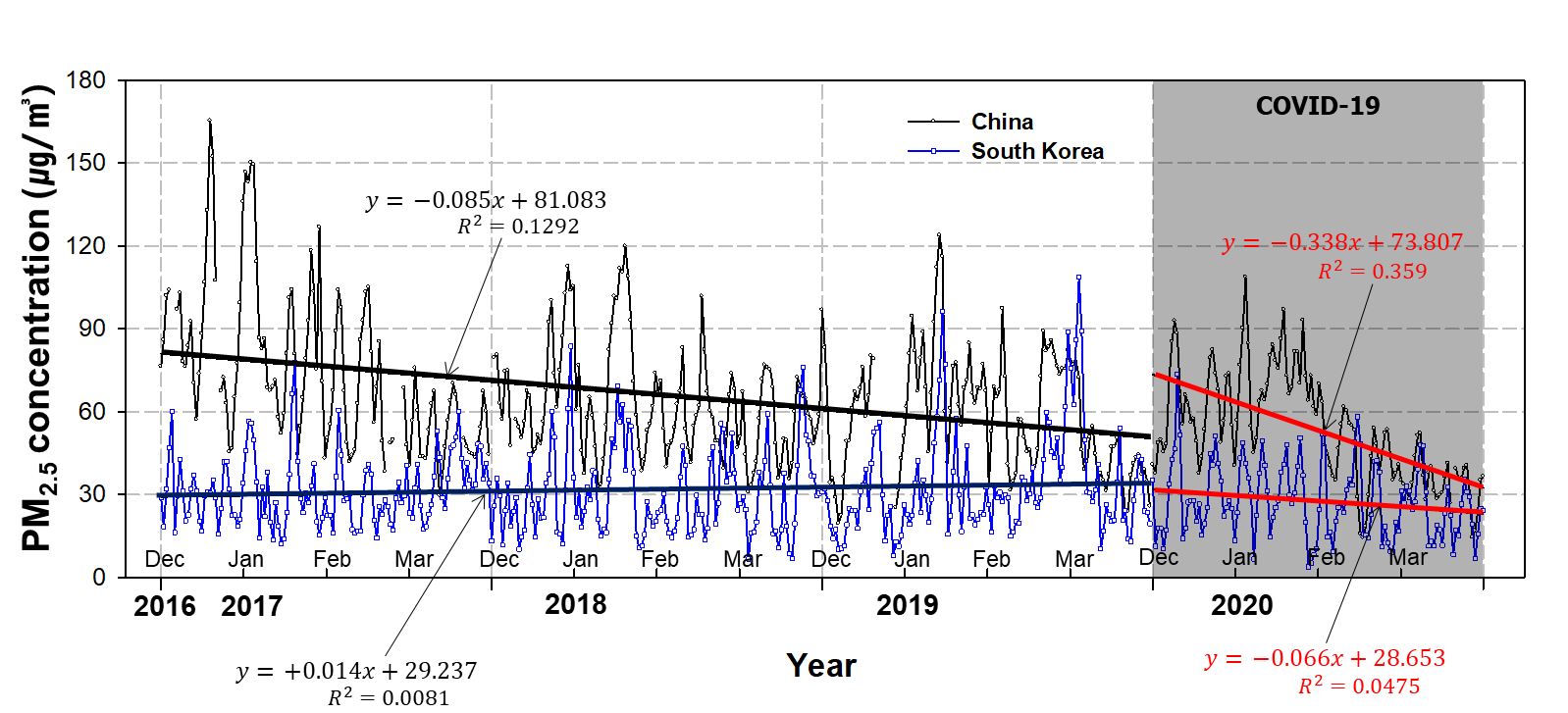


***Study regions***

Fig. S2 Study areas for Northeast Asia, including China and South Korea. China was divided into three regions (Northern, Central and Southern China). For South Korea, Seoul Metropolitan Area (SMA), a densely populated area including Seoul was separated. The map was generated using Interactive Data Language version 8.7.0 (Harris Geospatial Solutions, <http://harrisgeospatial.com>) with Global Administrative Areas (<http://gadm.org>) map data.


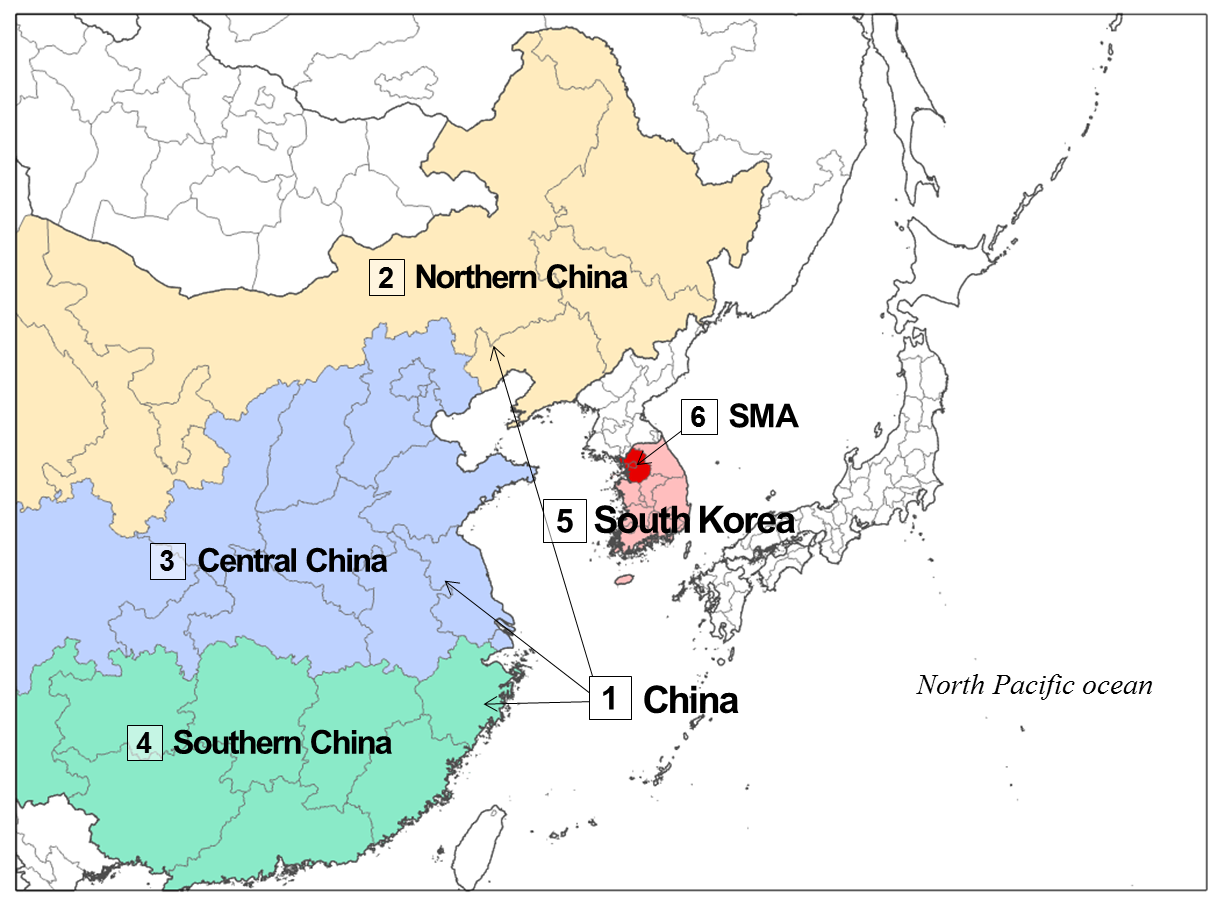


***Model performance evaluations during the non-COVID-19 and the COVID-19 periods***

For simulated meteorological factors for China and Korea, observations for the non-COVID-19 and COVID-19 periods showed similar agreement between the two periods (Table S1, Figs. S2~4). For air temperature at 2 m, the range was MBE -0.81 ~ 0.39 ℃, RMSE 0.55 ~ 1.17 ℃, and R 0.97 ~ 0.99, and for 10 m wind speed, MBE -0.16 ~ 1.10 ms^-1^, RMSE 0.32 ~ 1.22 ms^-1^, and R 0.88 ~ 0.98, indicating that the observation-simulation agreement was relatively high. In contrast, in the case of PM_2.5_, there was a large difference in the observation-simulation agreement between the COVID-19 period and the non-COVID-19 period (R value for non-COVID-19 period in China and in South Korea: 0.88, 0.89, R for COVID-19 period in China and in South Korea: 0.81, 0.81). The difference between the simulated and observed PM_2.5_ concentrations during the COVID-19 period can also be confirmed in time series of daily mean PM_2.5_ concentration (Fig. S2). For the non-COVID-19 period, most of the Northeast Asia region showed negative MBE (China -3.32 ㎍/㎥, Korea -5.41 ㎍/㎥, observed concentration > simulated concentration), whereas for the COVID-19 period, the simulated PM_2.5_ concentrations showed higher MBE value than those for the non-COVID-19 period (China +7.33 ㎍/㎥, Korea +1.62 ㎍/㎥, observed concentration < simulated concentration).

Table S1. Statistical verification of simulated daily mean meteorological factors (2 m temperature and 10 m wind speed) and PM_2.5_ concentrations for the Non-COVID-19 and COVID-19 periods. The meteorological data used for statistical verification were MADIS data from 35 points in China and 16 points in Korea. For PM_2.5_ concentration, the air quality monitoring data from 1,495 sites in China and 287 sites in Korea were used.

[a] Non-COVID-19 period (2016-2018)

| Region | Air temperature | | | Wind speed | | | PM_2.5_ | | |
| --- | --- | --- | --- | --- | --- | --- | --- | --- | --- |
|  | MBE^a^ | RMSE^b^ | R^c^ | MBE | RMSE | R | MBE | RMSE | R |
| China | 0.35 | 0.61 | 0.99 | 0.48 | 0.53 | 0.93 | -3.32 | 12.07 | 0.88 |
| North China | -0.30 | 1.02 | 0.98 | -0.16 | 0.54 | 0.91 | -22.92 | 26.31 | 0.78 |
| Central China | 0.39 | 0.80 | 0.99 | 0.06 | 0.32 | 0.93 | 5.87 | 15.14 | 0.90 |
| South China | 0.23 | 0.89 | 0.97 | 1.10 | 1.16 | 0.92 | -7.49 | 17.32 | 0.57 |
| South Korea | -0.74 | 1.07 | 0.99 | 0.25 | 0.38 | 0.98 | -5.41 | 9.10 | 0.89 |
| SMA | 0.19 | 1.17 | 0.98 | 0.39 | 0.60 | 0.94 | -6.57 | 12.26 | 0.85 |

[b] COVID-19 period (2019)

| Region | Air temperature | | | Wind speed | | | PM_2.5_ | | |
| --- | --- | --- | --- | --- | --- | --- | --- | --- | --- |
|  | MBE^a^ | RMSE^b^ | R^c^ | MBE | RMSE | R | MBE | RMSE | R |
| China | -0.38 | 0.55 | 0.99 | 0.49 | 0.53 | 0.93 | 7.33 | 12.32 | 0.81 |
| North China | -0.32 | 0.97 | 0.99 | 0.10 | 0.47 | 0.92 | -14.98 | 21.24 | 0.83 |
| Central China | 0.25 | 0.71 | 0.99 | 0.21 | 0.36 | 0.93 | 18.81 | 22.04 | 0.84 |
| South China | -0.81 | 1.07 | 0.98 | 0.88 | 0.94 | 0.91 | 1.31 | 12.81 | 0.51 |
| South Korea | -0.60 | 0.95 | 0.98 | 0.66 | 0.80 | 0.93 | 1.62 | 7.51 | 0.81 |
| SMA | 0.03 | 0.95 | 0.97 | 0.10 | 1.22 | 0.88 | 0.91 | 9.43 | 0.78 |

^a^ MBE: mean bias error

^b^ RMSE: root mean square error

^c^ R: Correlation coefficient

Fig. S3. Comparison between the observed and simulated PM_2.5_ concentrations in (a) China and (b) South Korea during 2016~2019. The black line and blue dots denote the simulated and observed PM_2.5_ concentrations, respectively.

| (a) China  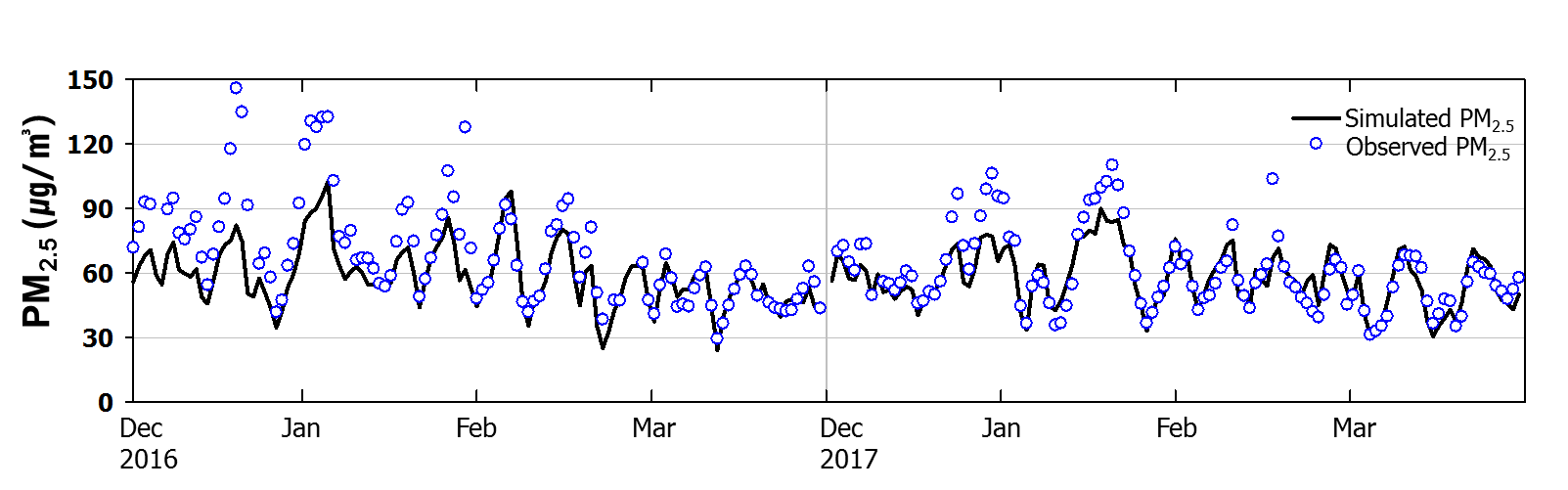 |
| --- |
| 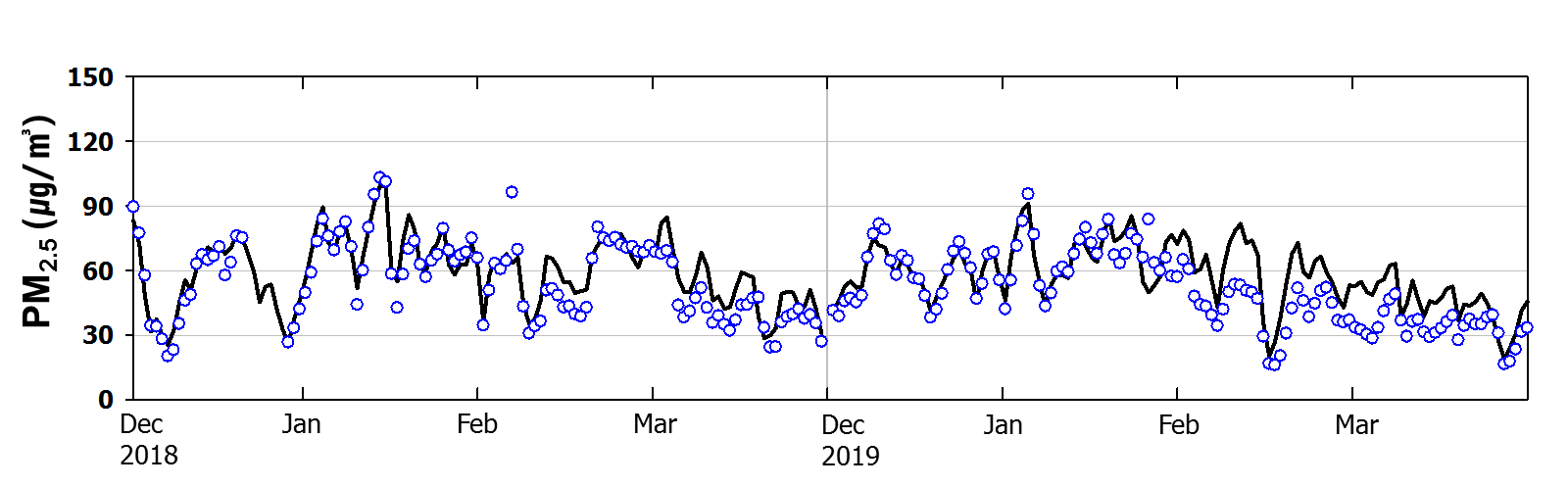 |
| (b) South Korea  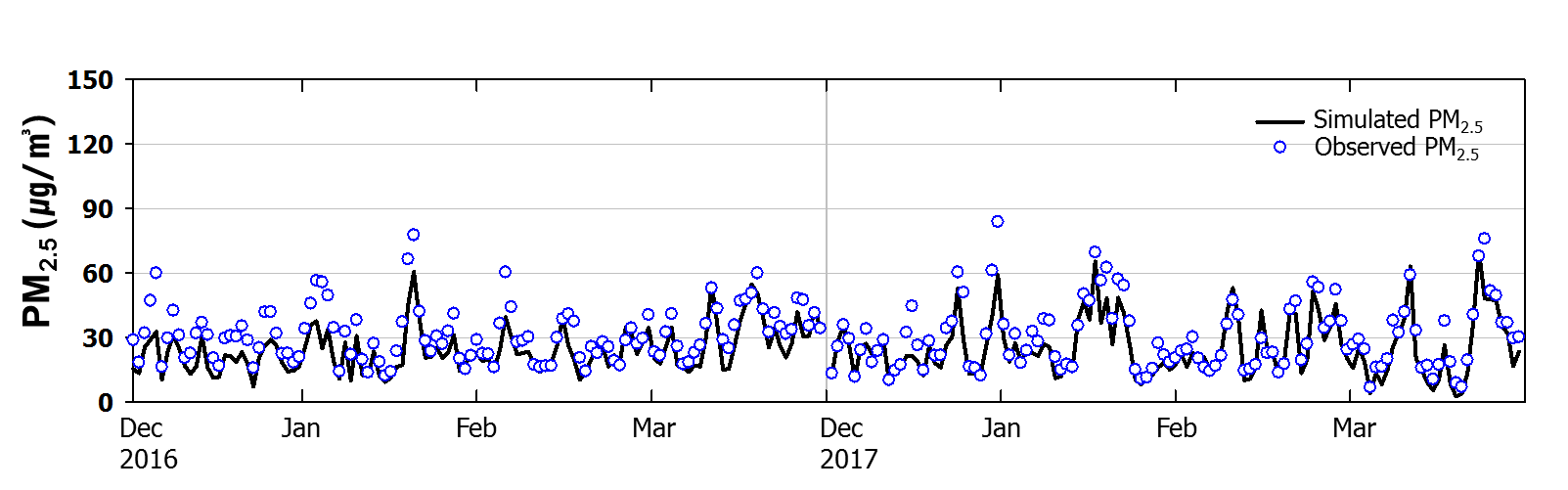 |
| 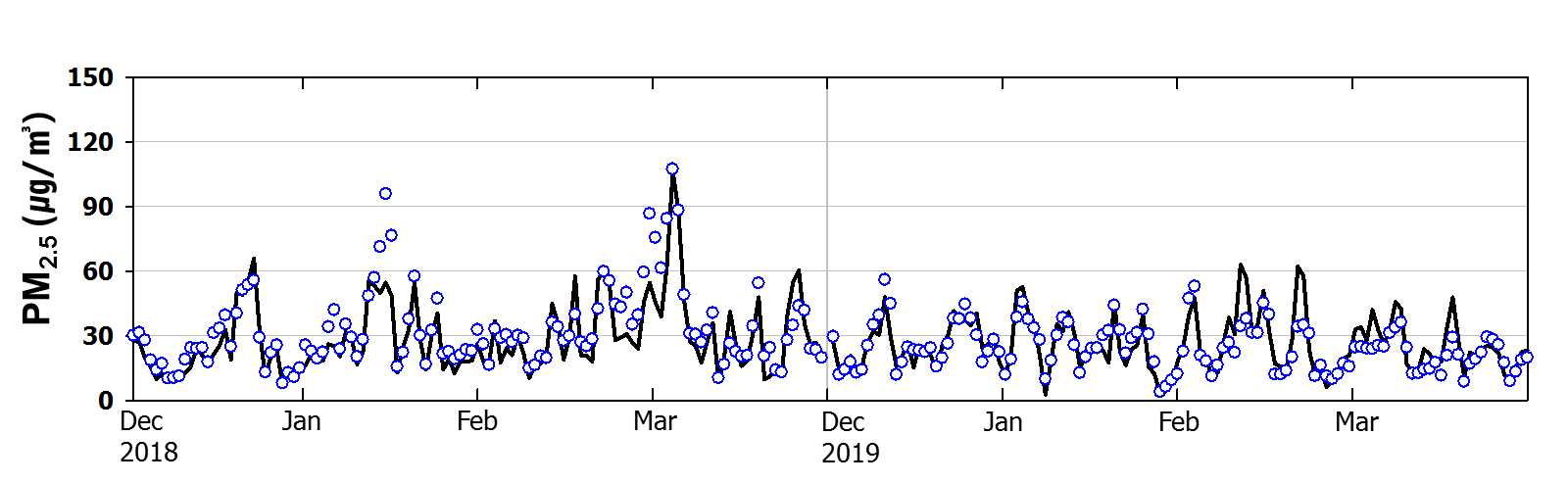 |

Fig. S4. Comparison between the observed and simulated air temperature in (a) China and (b) South Korea during 2016~2019. The black line and blue dots denote the simulated and observed air temperatures, respectively.

| (a) China  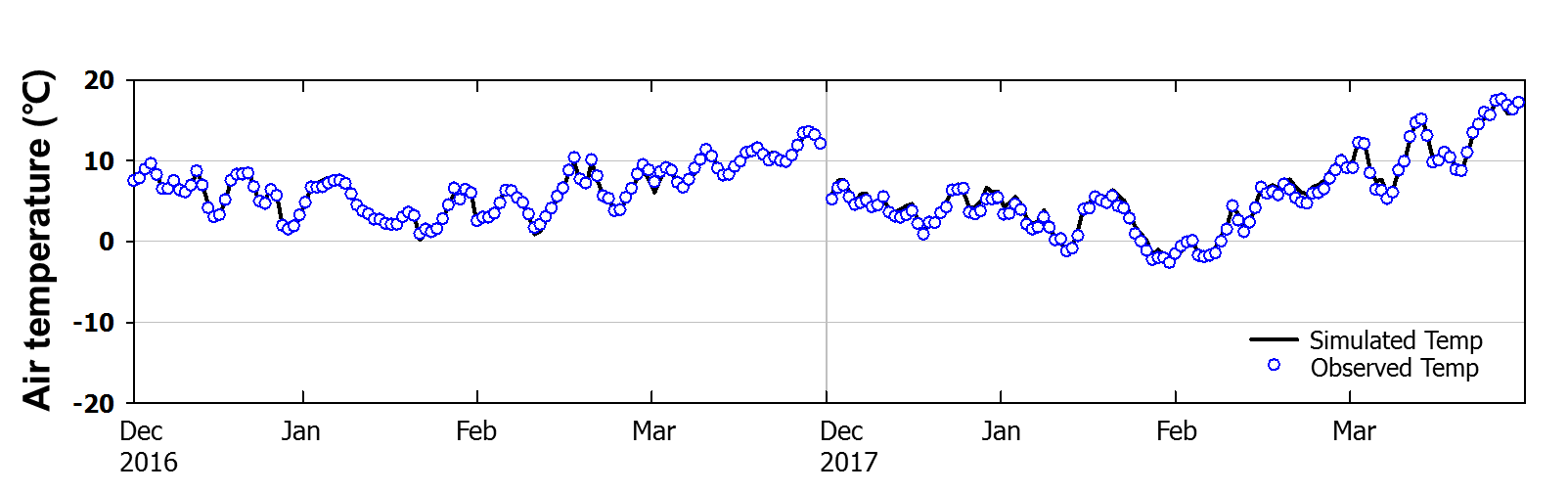 |
| --- |
| 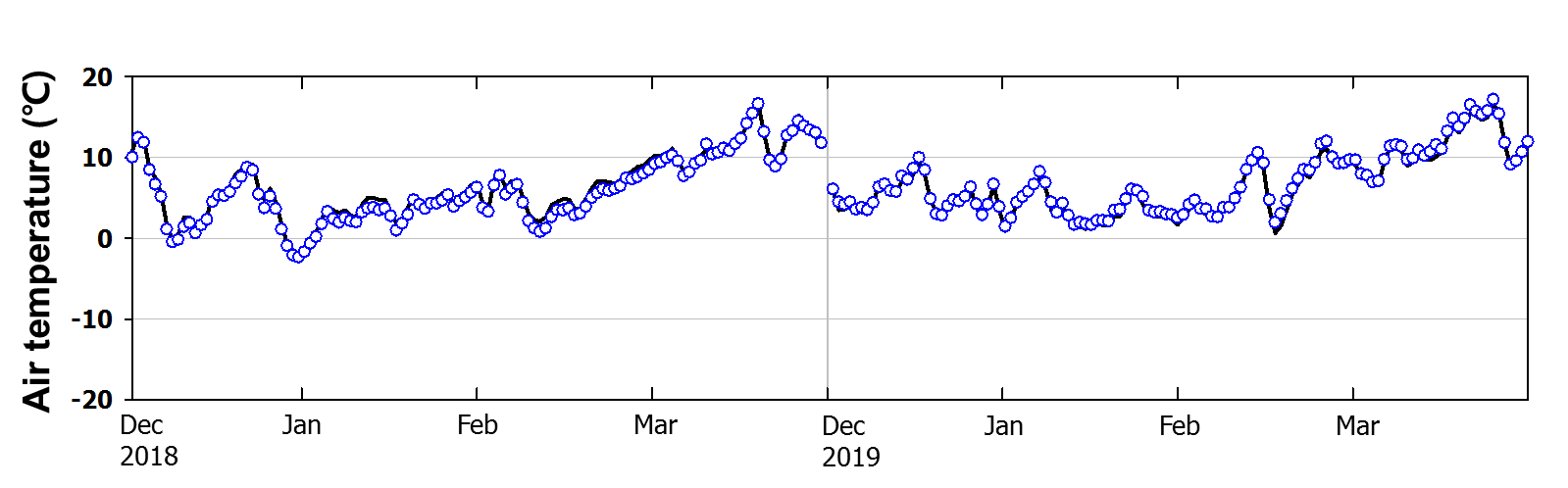 |
| (b) South Korea  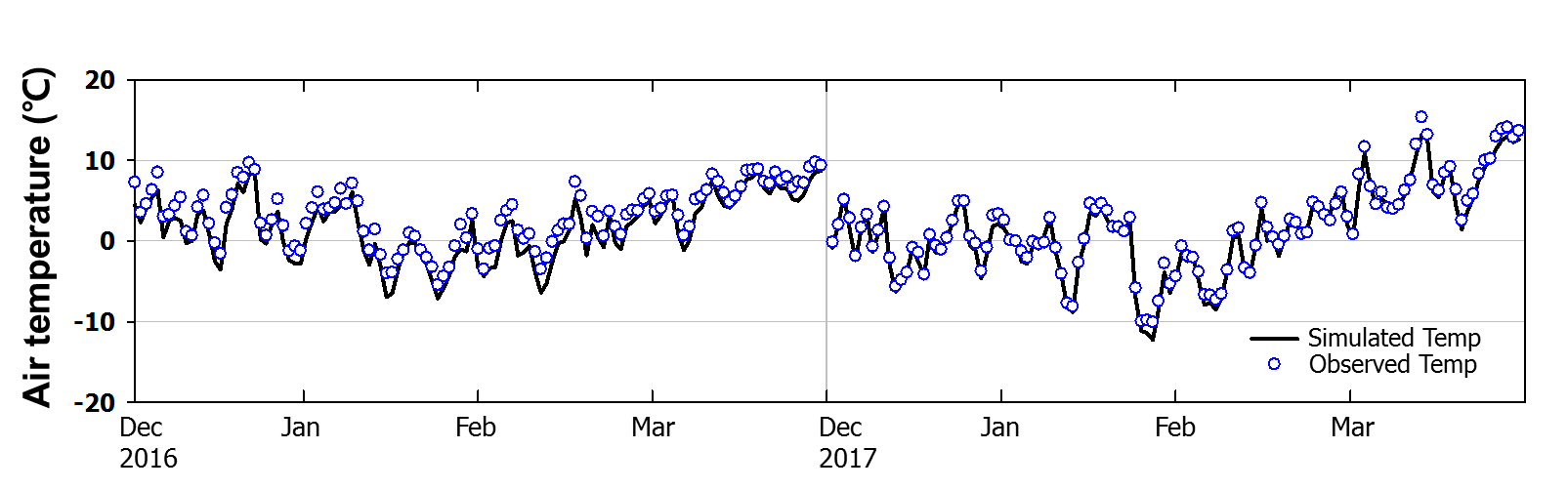 |
| 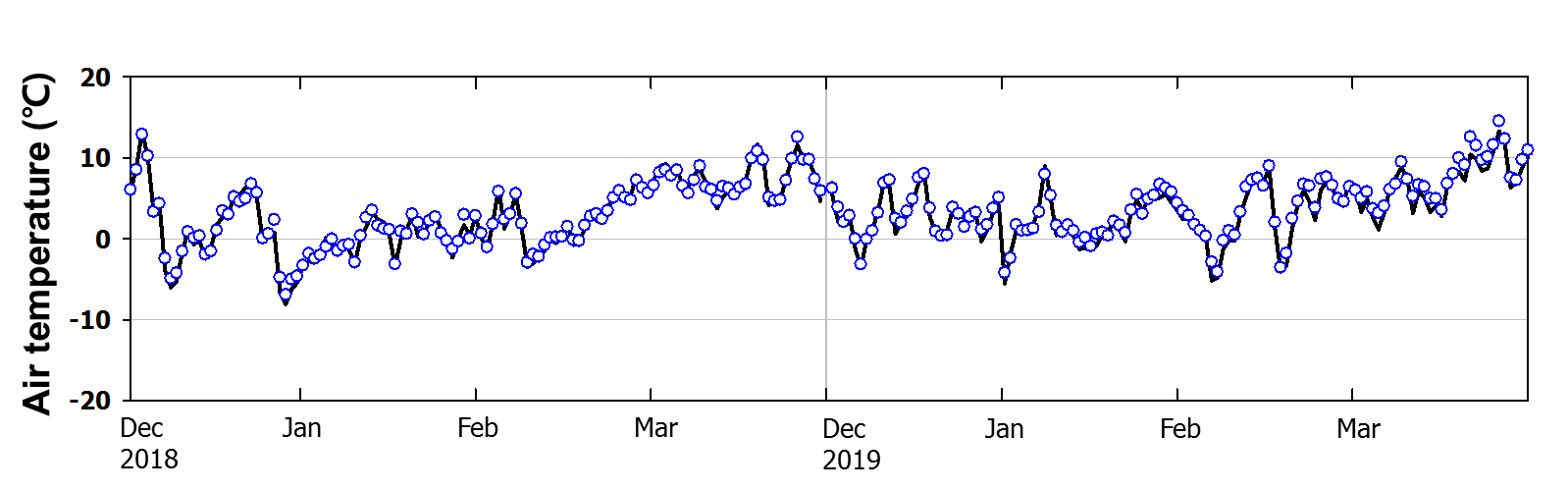 |

Fig. S5. Comparison between the observed and simulated wind speeds in (a) China and (b) South Korea during 2016~2019. The black line and blue dots denote the simulated and observed wind speeds, respectively.

| (a) China  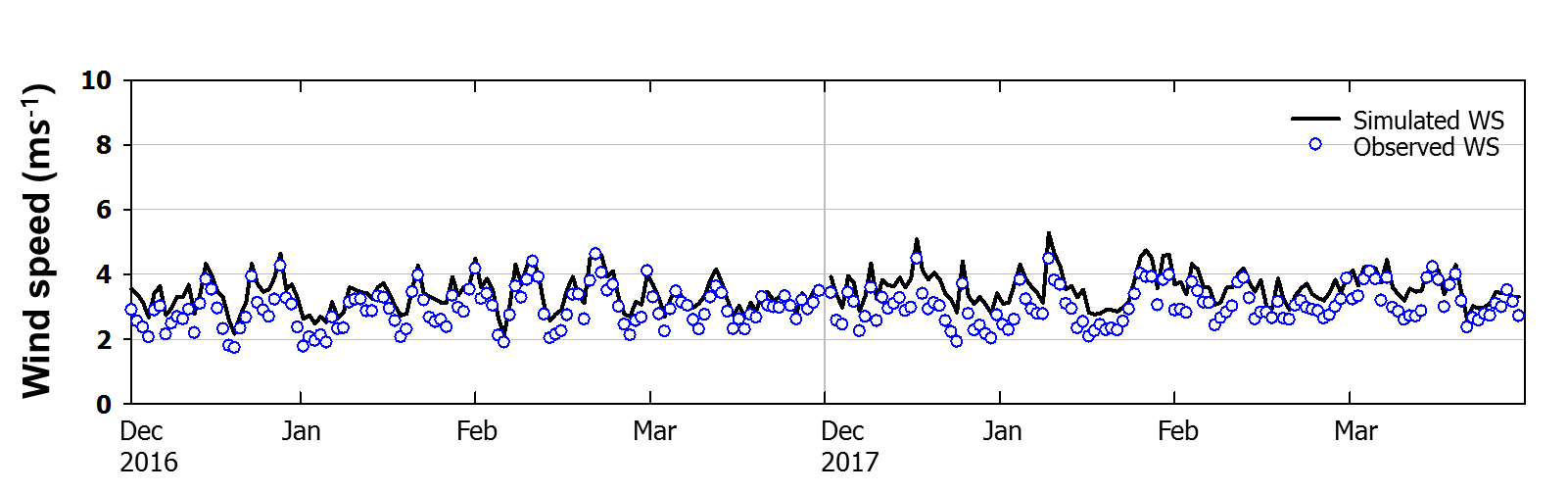 |
| --- |
| 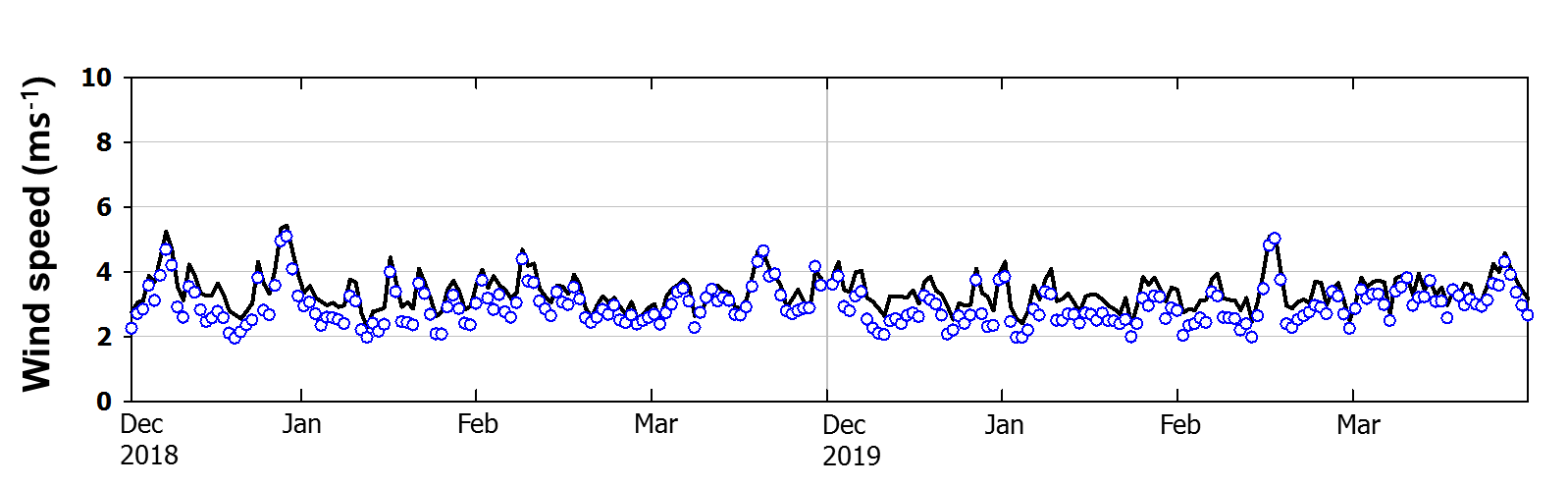 |
| (b) South Korea  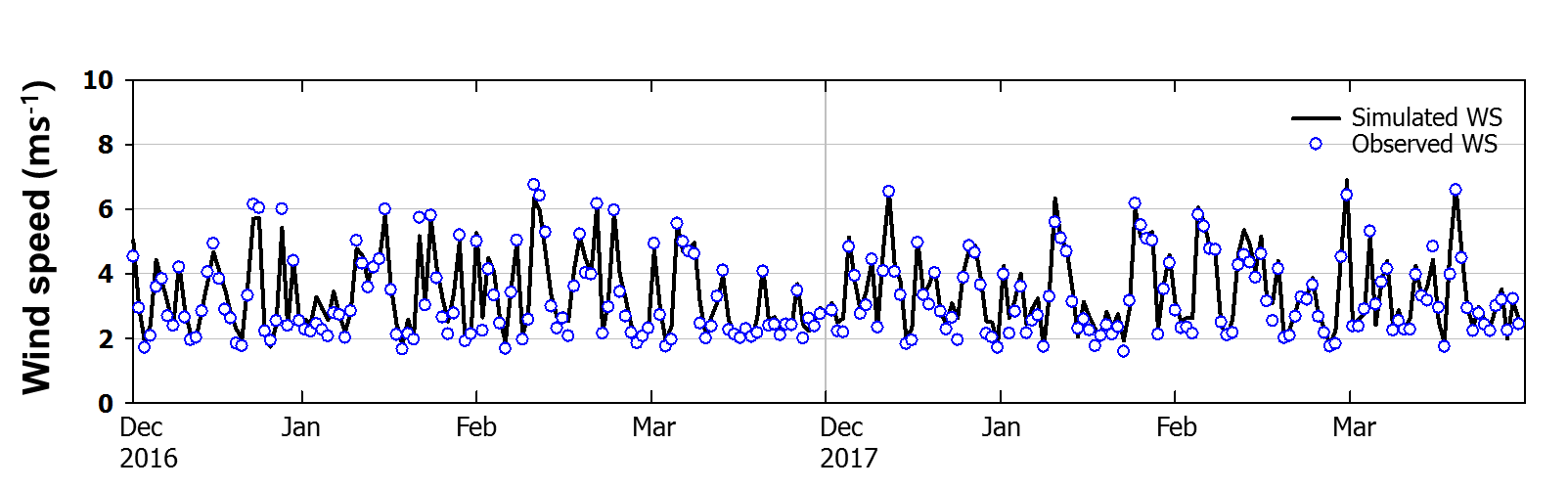 |
| 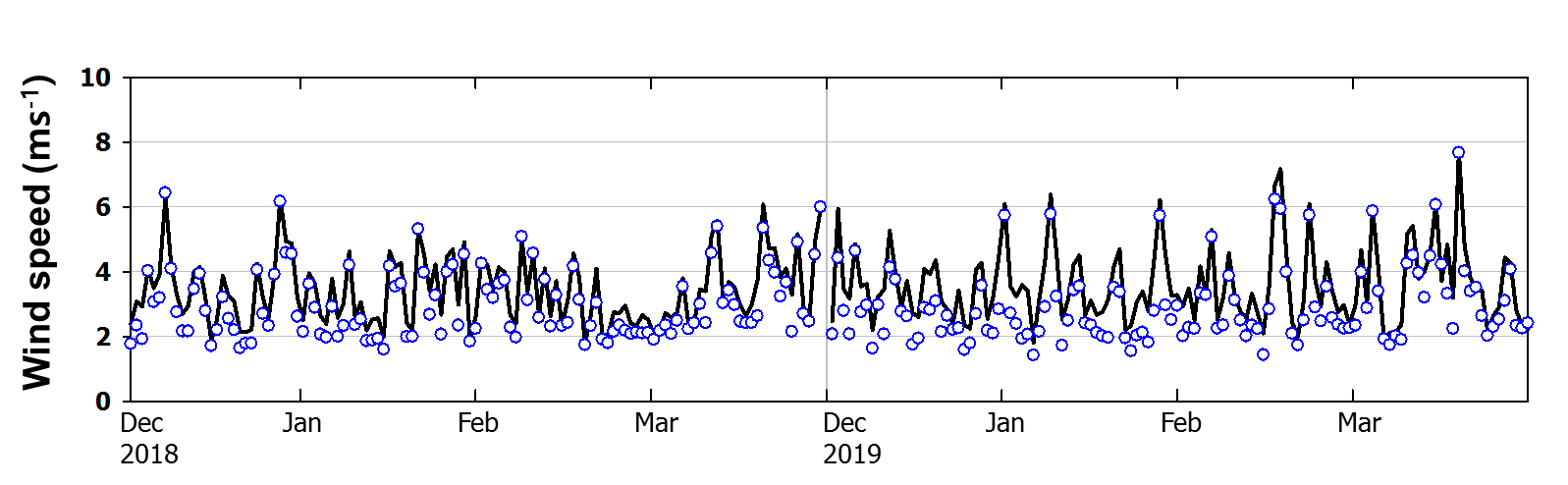 |

***Meteorological conditions compared between the COVID-19 period and the non-COVID-19 period in Northeast Asia***

Fig. S6. Spatial distributions of the averaged (a) 2 m Relative humidity and (b) 10 m wind speed in the Non-COVID-19 periods, the COVID-19 period and the difference between the Non-COVID-19 and COVID-19 periods. The maps were generated using the NCAR Command Language (version 6.2.1) Boulder, Colorado: UCAR/NCAR/CISL/TDD. http://dx.doi.org/10.5065/D6WD3XH5.

(a) 2 m relative humidity

| December and January  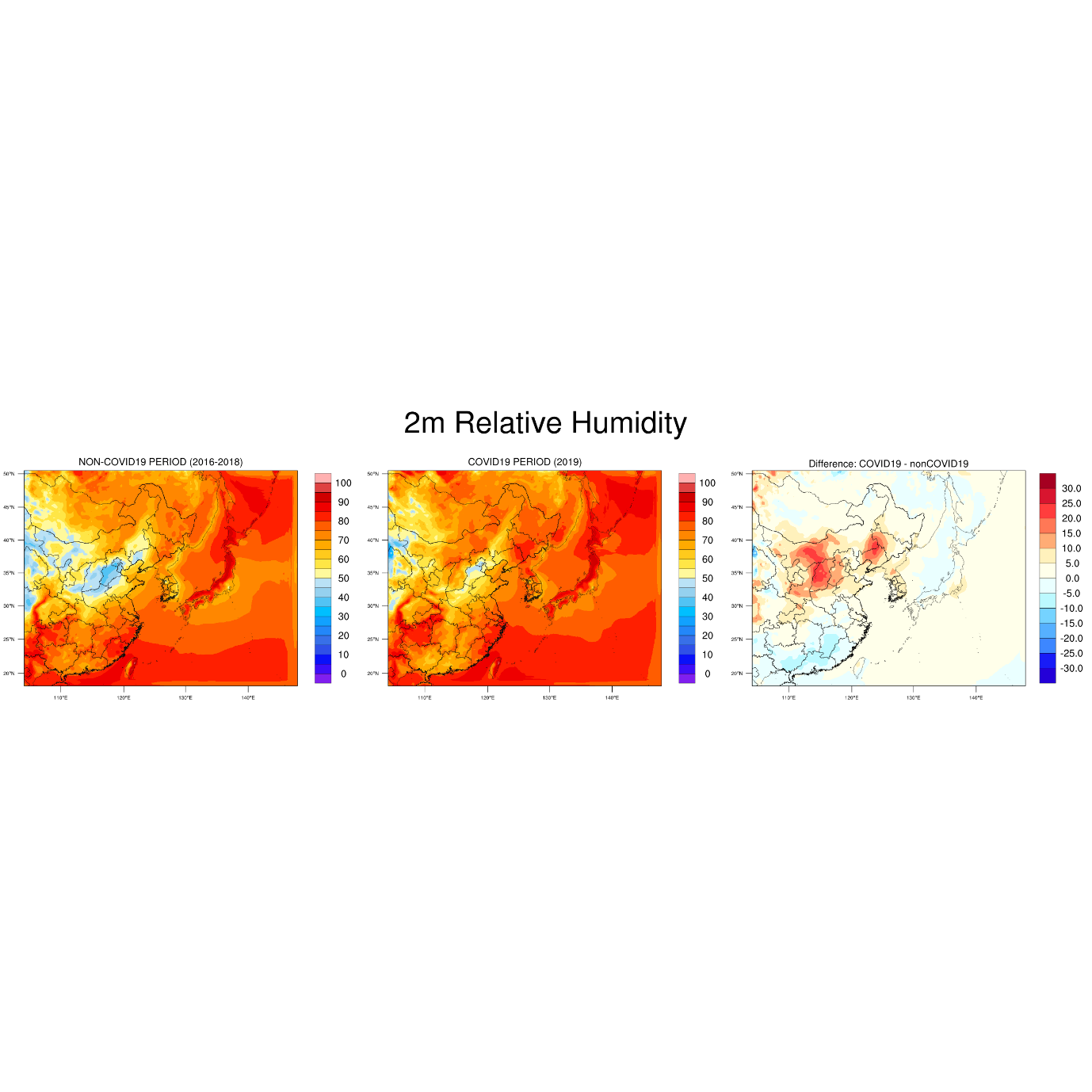 |
| --- |
| February and March  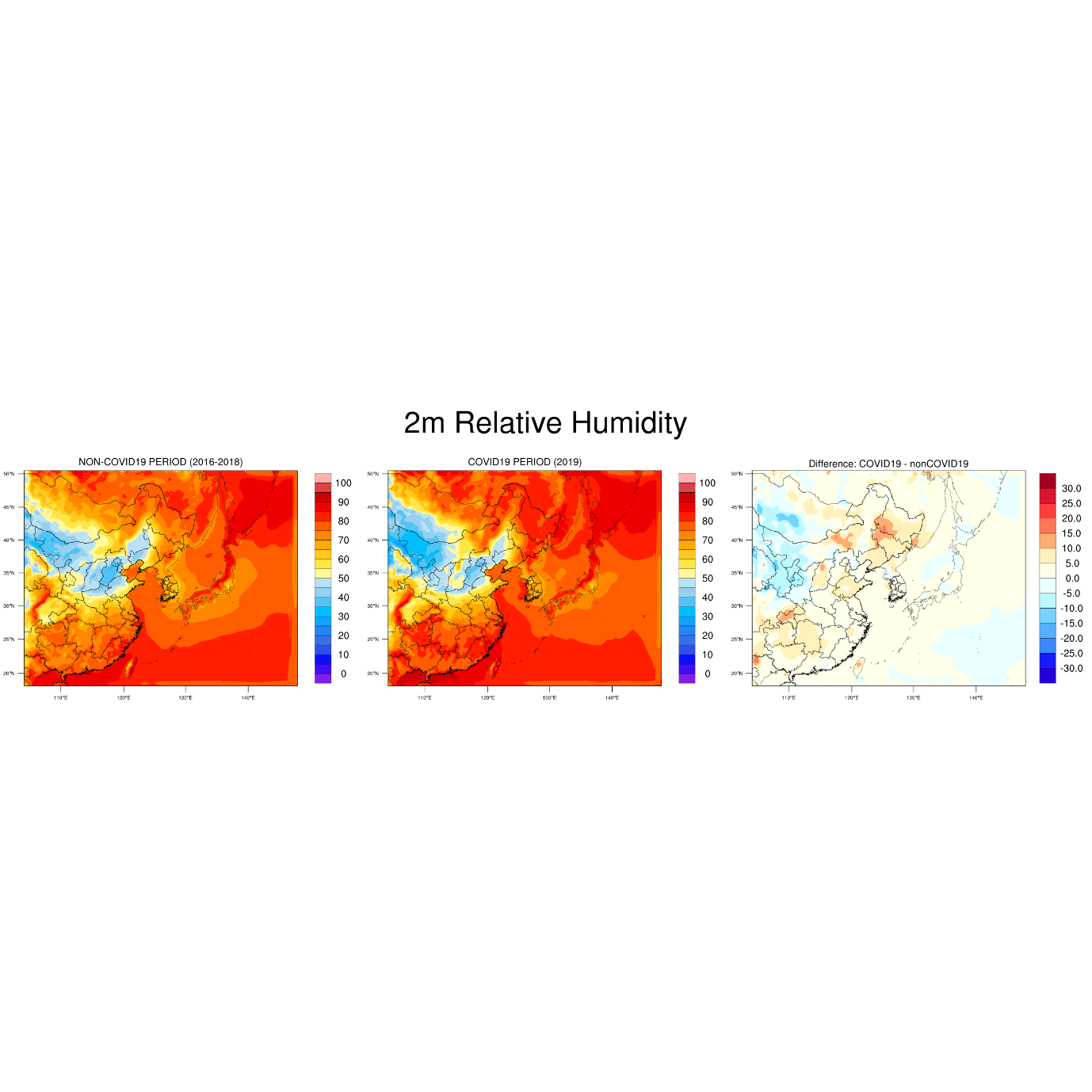 |

(b) 10 m wind speed

| December and January  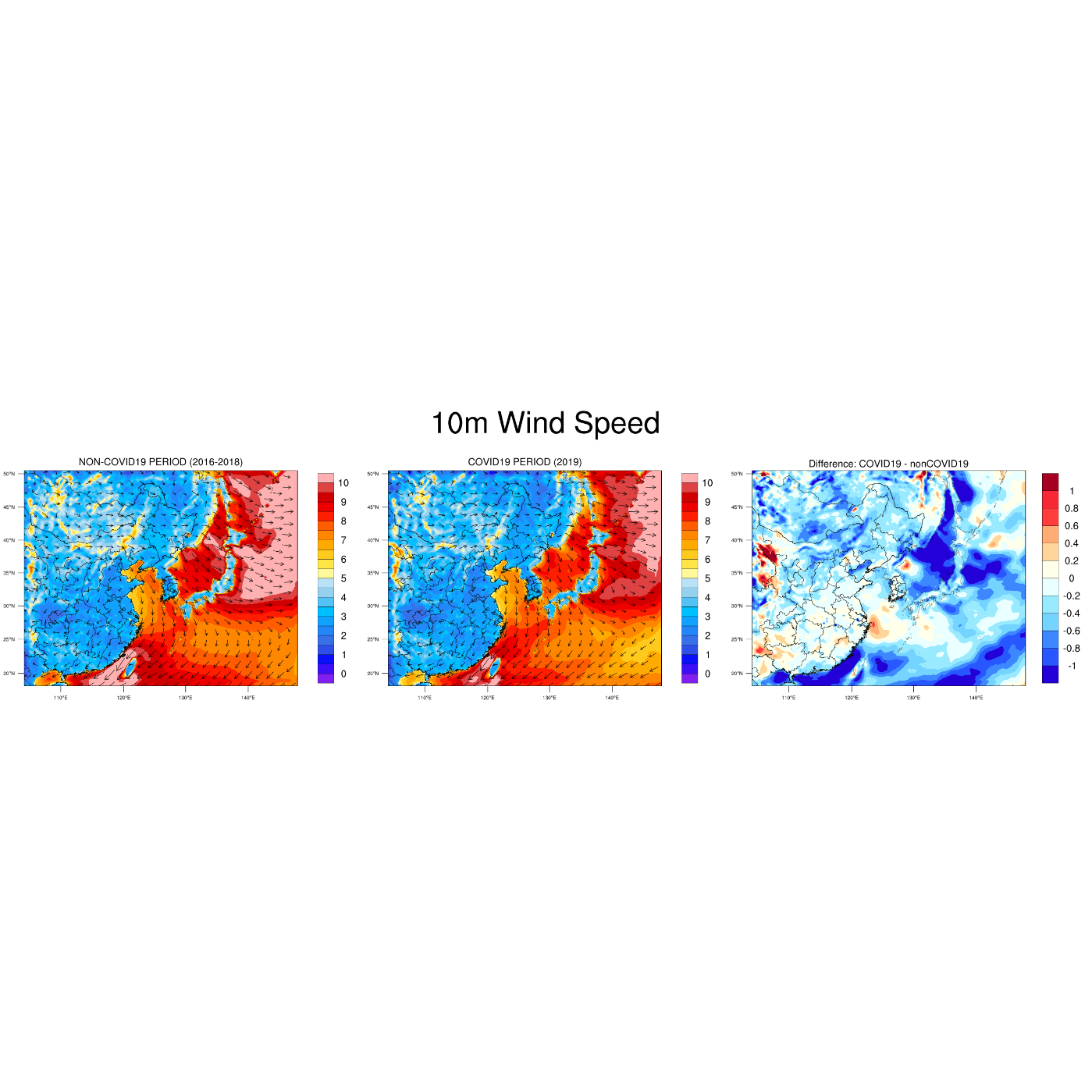 |
| --- |
| February and March  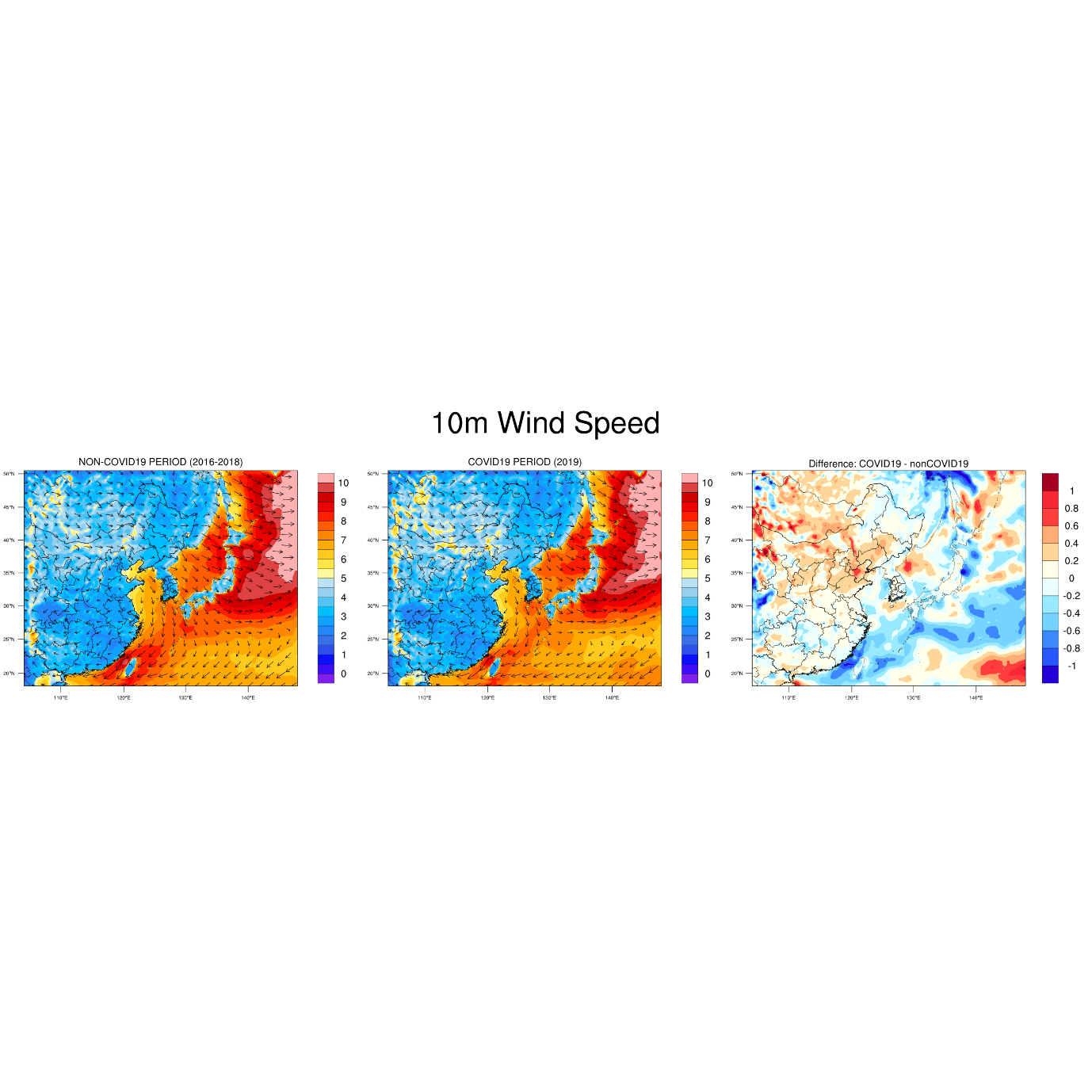 |

***Spatial differences in PM_2.5_ concentration reduction during the COVID-19 period***

There were regional differences in PM_2.5_ concentration changes from February to March 2020, when various control measures on external activities of public due to COVID-19 were in full operation (Fig. S7a). Compared with the previous year (February to March 2019), the concentration change of PM_2.5_ in China during the COVID-19 period were -15.6 ㎍/㎥ (-29%), -16.1 ㎍/㎥ (-28%), -7.1 ㎍/㎥ (-19%), respectively, for the northern, central and southern regions, indicating the smallest concentration change in the southern region (See Fig. S2 for the regional division). South Korea had a PM_2.5_ concentration change of -13.5 ㎍/㎥ (-37%) during the COVID-19 period compared with that observed in the previous year, and the Seoul Metropolitan Area (SMA), showed the change at -14.9 ㎍/㎥, indicating a greater PM _2.5_ reduction by 1.4 ㎍/㎥. Aerosol Optical Depth (AOD) images (Fig. S7b) taken from Moderate Resolution Imaging Spectroradiometer (MODIS) during the same period showed reduced concentration of aerosol concentrations in the atmosphere during the COVID-19 period in the Northeast Asian region, including seas where no ground observations exist. In particular, during the period from February to March 2019, the AOD (0.5 or higher), whose high values were mainly observed on the eastern coast of China, the Yellow Sea, and the western coastal areas of South Korea, fell to 0.4 or lower, except for coastal cities, during the COVID-19 period, representing clear decrease of AOD.

Fig. S7 (a) Mean spatial distribution of PM_2.5_ by administrative district observed in China and South Korea in February and March 2019 and 2020 and (b) AOD in Northeast Asia as observed in MODIS satellite images. The top panel shows the mean values from February to March 2019 before the COVID-19 outbreak, and the bottom panel shows the mean values from February to March 2020, the COVID-19 period. The maps were generated using Interactive Data Language version 8.7.0 (Harris Geospatial Solutions, <http://harrisgeospatial.com>) with Global Administrative Areas (<http://gadm.org>) map data.

| (a) |  | (b) |  |
| --- | --- | --- | --- |
| Surface PM_2.5_ (Feb ~ Mar 2019)  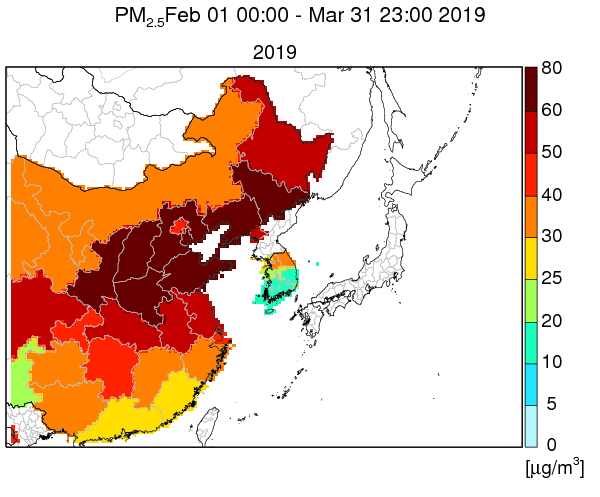 | 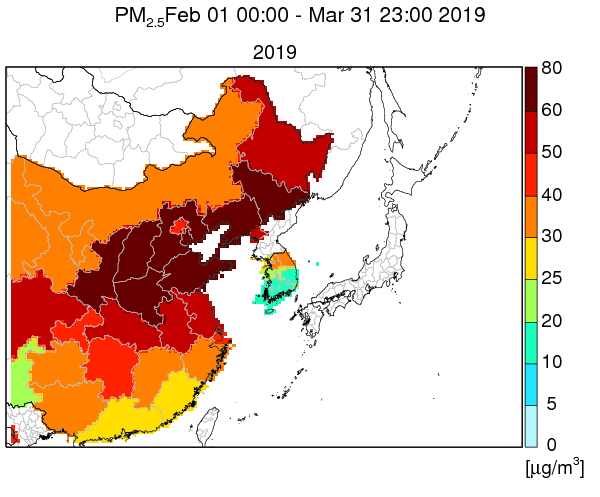 | MODIS AOD (Feb ~ Mar 2019)  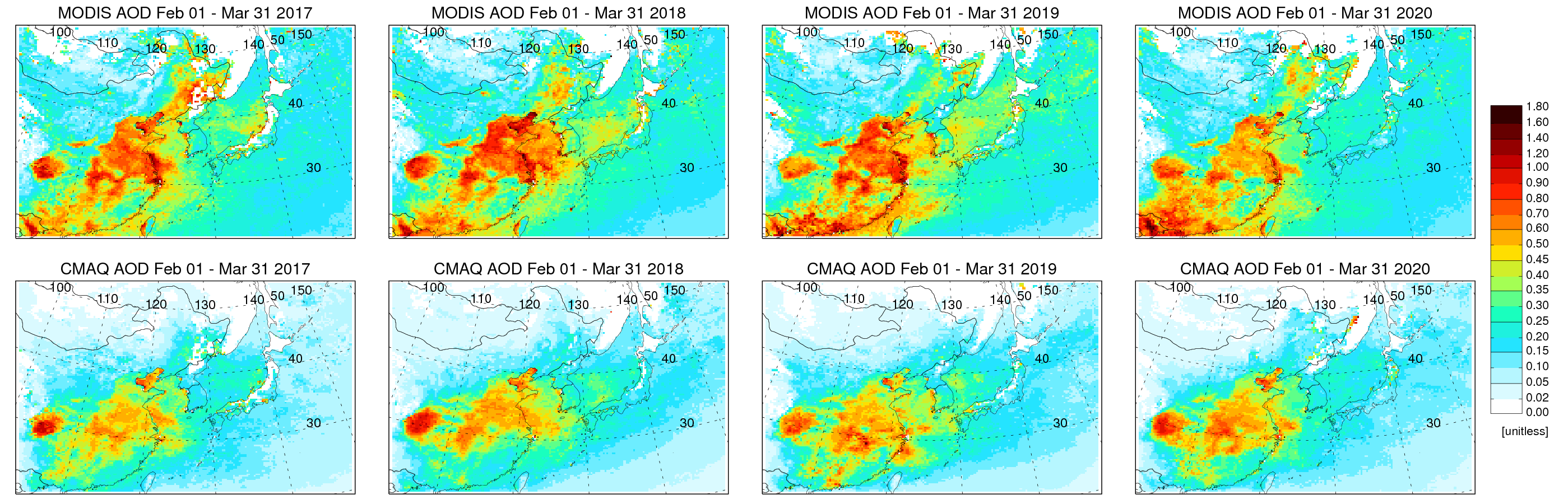 | 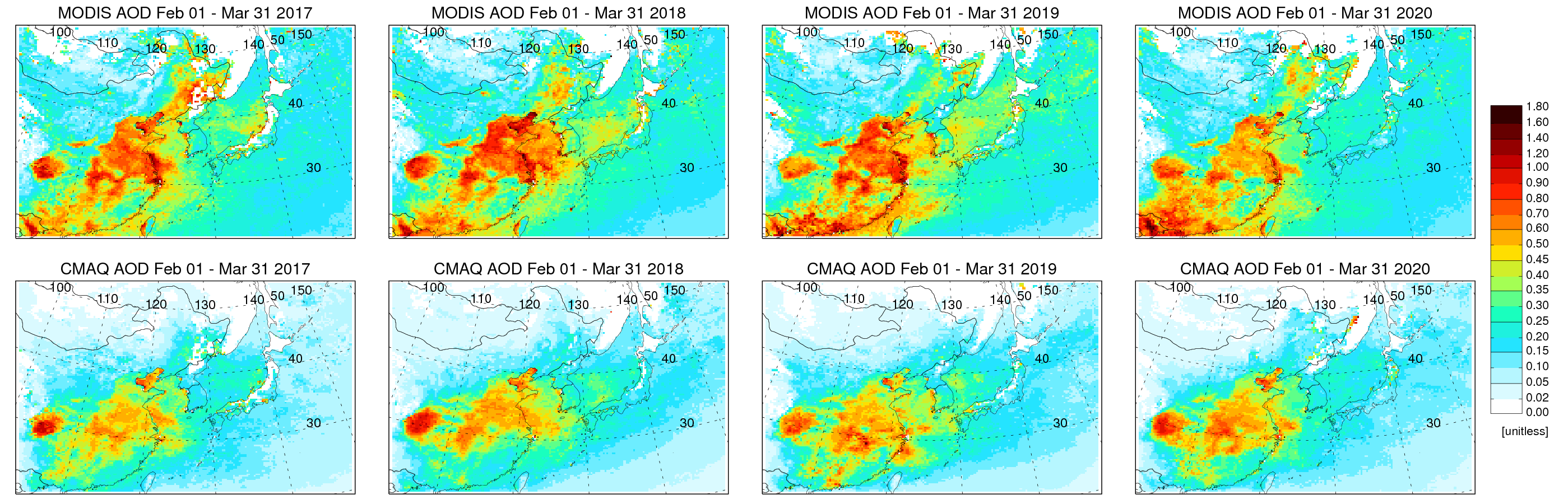 |
| Surface PM_2.5_ (Feb ~ Mar 2020)  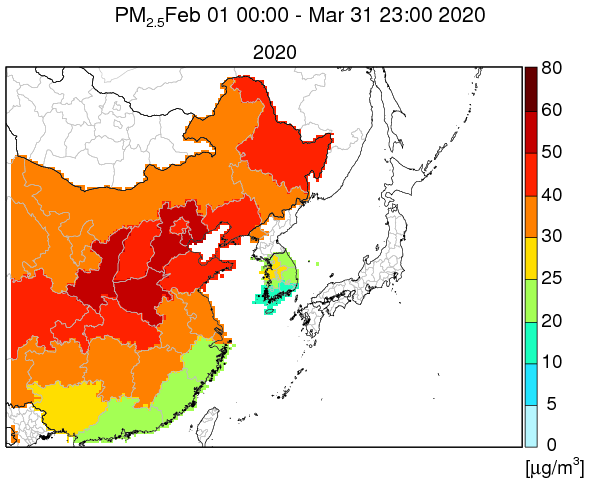 |  | MODIS AOD (Feb ~ Mar 2020)  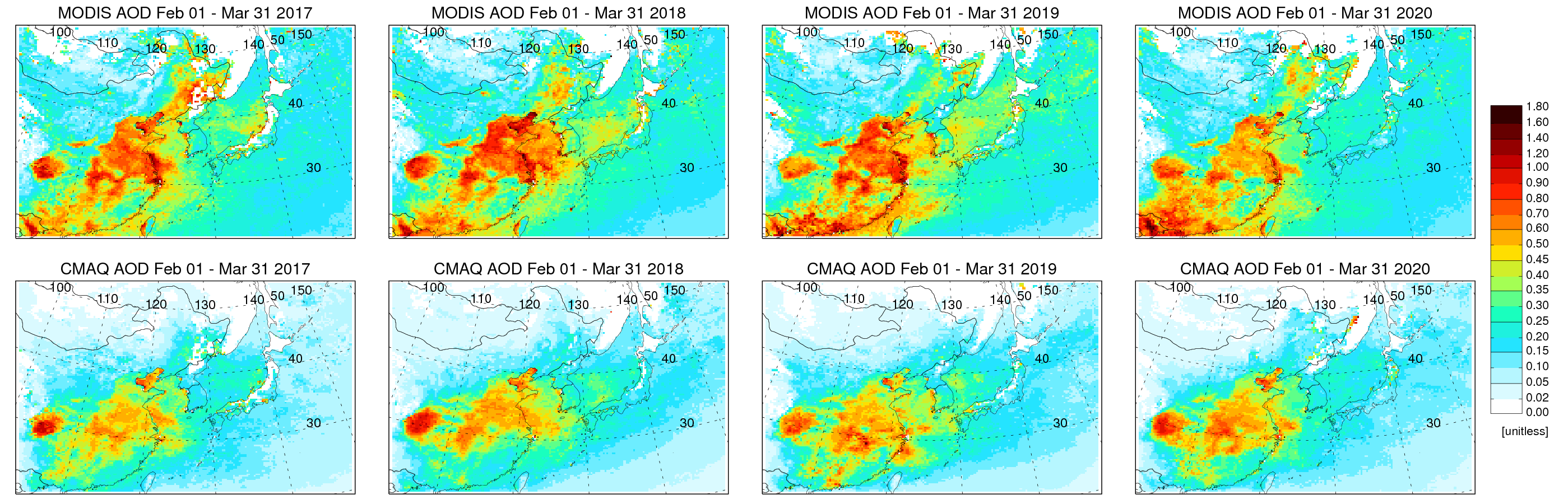 |  |
